# Sustainable Low-Carbon Cement: Performance Enhancement with Calcined Natural Pozzolans Through Compressive Strength, Porosity, and Microstructural Analysis

**DOI:** 10.3390/ma18081776

**Published:** 2025-04-13

**Authors:** Magnolia Soto Felix, Roger Ulisses Hernandez Zamora, Miguel Armando Avila Rubio, Caleb Carreño Gallardo, Jose Martin Herrera Ramirez

**Affiliations:** 1 Facultad de Ingenieria Culiacan, Universidad Autonoma de Sinaloa, Culiacan 80040, Mexico; 2 Facultad de Ingenieria Mochis, Universidad Autonoma de Sinaloa, Los Mochis 81223, Mexico; 3 Centro de Investigación en Materiales Avanzados, S.C. (CIMAV), Av. Miguel de Cervantes No. 120, Complejo Industrial Chihuahua, Chihuahua 31136, Mexico

**Keywords:** low-carbon cement, calcined natural pozzolans, compressive strength, microstructure, pozzolanic reaction

## Abstract

The global cement industry faces a critical challenge of reducing its substantial carbon footprint while maintaining material performance. Portland cement production significantly contributes to global CO_2_ emissions, necessitating innovative sustainable alternatives. This study evaluates the transformative potential of calcined natural pozzolans as a strategic approach to developing low-carbon cement. By systematically investigating the effects of calcined natural pozzolans derived from kaolinite and pyroclastic rocks on cement paste properties, the research demonstrates a promising pathway to environmentally efficient cement formulations. Utilizing advanced characterization techniques including XRD, TGA, SEM-EDX, and gas adsorption porosimetry, this study provides insights into hydration kinetics, compressive strength development, microstructural evolution, and porosity refinement. The results reveal that calcined natural pozzolans strategically enhance cement performance by accelerating hydration processes, improving compressive strength, and sophisticating microstructural characteristics. Notably, pastes incorporating pyroclastic rock pozzolans exhibited superior mechanical properties, with 28-day compressive strengths exceeding ordinary Portland cement by 35.2%. These findings not only validate the technical feasibility of natural pozzolan-based low-carbon cement but also underscore their potential to meaningfully reduce the construction industry’s environmental impact.

## 1. Introduction

Portland cement (PC), primarily composed of clinker, remains one of the most widely used construction materials due to its availability and cost-effectiveness. However, its production is a significant contributor to global CO_2_ emissions. In 2020, global cement production was approximately 4.2 Gt, with emissions reaching nearly 2.9 Gt of CO_2_, representing around 7–8% of total anthropogenic emissions. By 2023, production continued to increase, particularly in major cement-producing countries such as China and India, exacerbating its environmental impact [1]. To mitigate CO_2_ emissions associated with PC production, reducing clinker content through partial substitution with supplementary cementitious materials (SCMs), such as natural or calcined pozzolans, has proven to be an effective strategy. This approach decreases the carbon footprint while maintaining or even improving cementitious performance, ensuring compatibility with existing manufacturing processes [2,3,4,5].

Pozzolans are siliceous or siliceous–aluminous materials with little to no intrinsic cementitious properties. However, when finely ground and exposed to moisture, they react with calcium hydroxide (CH), a by-product of cement hydration, forming secondary hydration compounds that enhance the mechanical strength and durability of concrete. These materials, which can be classified as either natural or artificial, play a crucial role in improving the mechanical and durability properties of concrete [6,7]. Artificial pozzolans, such as fly ash (FA) and silica fume (SF), are industrial by-products and those that require less energy for their production than PC such as metakaolin (MK). 

FA, composed of fine, powdery particles primarily spherical in shape, is the predominant component of coal ash, formed during the combustion of pulverized coal in power plants, and enhances the workability, long-term strength, and durability of concrete [8]. In contrast, SF, a by-product of the silicon and ferrosilicon alloy industries, consists of ultra-fine, amorphous silica particles with a highly irregular and agglomerate morphology, significantly increasing its pozzolanic reactivity. Similarly, MK, produced through the calcination of kaolin clay, is a highly reactive pozzolan that improves workability, mechanical strength, and sulfate resistance in cementitious materials [9].

The availability of artificial pozzolanic by-products such as FA and SF has declined precipitously in recent years. This scarcity stems from multiple factors: the systematic decommissioning of coal-fired power plants, implementation of advanced clean combustion technologies, and intensifying cross-sector competition that severely constrains FA supply chains. Concurrently, the silicon and ferrosilicon alloy industries have experienced diminished demand, while metallurgical recovery processes have undergone significant transformations, collectively triggering a substantial reduction in SF availability. These converging market forces have created unprecedented supply constraints for these valuable pozzolanic materials [10].

Against this backdrop of scarcity, natural and calcined pozzolans—particularly MK—have emerged as compelling and environmentally superior alternatives. MK, derived through the controlled thermal processing of kaolinite clay, requires substantially less energy input than traditional clinker production methods. This material not only diminishes the environmental footprint associated with cement manufacturing but simultaneously enhances concrete’s mechanical performance and long-term durability characteristics. Notably, MK incorporation has demonstrated remarkable efficacy in suppressing alkali–silica reaction (ASR), a deleterious phenomenon responsible for the concrete expansion and structural cracking [11]. These alternative SCMs offer dual benefits: they represent more sustainable options for reducing cement’s environmental impact while serving as practical and effective substitutes in regions experiencing acute shortages of conventional SCMs like fly ash, slag, and silica fume [12].

Metakaolin is produced through the calcination of kaolinite at temperatures between 500 and 800 °C. This process dehydroxylates kaolinite, disrupting its crystalline structure and generating an amorphous phase with high pozzolanic activity, comparable to SF [13,14]. Its incorporation enhances early-age compressive strength, reinforcing its potential for sustainable cementitious applications [15]. This improvement is attributed to both the filler effect and pozzolanic reactions, leading to the formation of calcium silicate hydrate (C-S-H), calcium aluminate hydrates (C_4_AH_13_ and C_3_AH_6_), and calcium aluminosilicate hydrate (C_2_ASH_8_) phases [16]. The reactivity of MK depends on the kaolinite source, calcination parameters, and particle fineness, all of which influence its effectiveness in low-carbon cement formulations [17].

Studies have reported up to a 24% increase in compressive strength with a 15% MK replacement, while replacements of 10–20% have demonstrated significant improvements in strength and durability compared to conventional cement mixtures [18,19]. However, higher MK content can negatively impact workability, increase water demand, and shorten setting time, limiting its optimal replacement level to approximately 20% [20]. These considerations underscore the importance of optimizing MK dosage to balance performance and practical applicability in low-carbon cement formulations.

In addition to MK, pyroclastic rock, a natural pozzolan formed during explosive volcanic eruptions [21], has received comparatively less scientific attention despite demonstrating remarkable potential due to its inherent pozzolanic reactivity and abundant regional availability throughout volcanic territories. These materials, primarily composed of amorphous silica, quartz, and feldspar, develop significant pozzolanic activity when subjected to calcination, which increases surface area and disrupts crystalline phases [22]. This process enhances their reactivity by increasing surface area and disrupting crystalline phases, improving their performance in cementitious systems [23]. Studies on similar volcanic materials, such as pumicite and zeolite, have demonstrated improved compressive strength and reduced porosity when incorporated into cement blends [24].

In addition, recent studies have examined the pozzolanic behavior of calcined pyroclastic rocks, with particular emphasis on volcanic tuffs containing high concentrations of amorphous silica, volcanic glass, and zeolitic phases. Research conducted on pyroclastic deposits from southern Europe has confirmed their pozzolanic properties through strength activity index assessments and mineralogical analyses. These studies report significant enhancements in compressive strength at replacement levels ranging between 10% and 40% [25]. Moreover, recent comprehensive evaluations of natural pozzolans containing varying proportions of volcanic glass and zeolites have demonstrated that both mineralogical composition and thermal activation protocols substantially influence their reactivity profiles [26].

Recent work on alternative binders has demonstrated that calcined pyroclastic rocks can also perform well in alkali-activated cementitious formulations. Mortars made with volcanic powders combined with recycled demolition waste achieved compressive strengths exceeding 40 MPa, thereby validating their considerable potential for implementation in sustainable construction practices without PC dependency [27]. Other investigations have focused on the long-term chemical interaction between cement and volcanic substrates under hydrothermal conditions. Studies examining the interfacial transition zone between cementitious materials and pyroclastic rocks have shown that phase formation mechanisms, carbonation rates, and silica enrichment patterns significantly influence durability performance under elevated temperature regimes and in CO_2_-rich environments [28]. These findings complement previous research and reinforce the technical and environmental feasibility of incorporating regionally available volcanic materials in the development of next-generation sustainable cement alternatives.

Despite their promising characteristics, pyroclastic rocks exhibit significant compositional and physical variability, which can influence their cementitious performance. Their use as SCMs poses challenges such as reduced workability, slower strength development, and increased water demand. Additionally, understanding their mineralogical and physical properties is essential to optimize their reactivity and ensure their effectiveness in low-carbon cement applications. 

While most research has focused on industrial by-product pozzolans, calcined kaolin, and natural pyroclastic rocks provide a sustainable alternative by reducing clinker dependence and CO_2_ emissions. Recent data show cement production has continued to rise significantly in recent years, with corresponding CO_2_ emissions now accounting for a substantial and growing portion of global anthropogenic emissions. This underscores the urgency of developing effective SCMs. The natural pozzolans studied in this work demonstrate promising characteristics, with pyroclastic rock samples exhibiting high SiO_2_ + Al_2_O_3_ + Fe_2_O_3_ content and MK showing excellent pozzolanic activity. Their local availability not only minimizes transportation-related impacts but also facilitates their integration into cementitious materials. 

Unlike previous studies that examined these materials in isolation or with limited characterization, our novel approach provides a comprehensive comparative analysis of both materials using advanced techniques including X-ray diffraction (XRD), thermogravimetric analysis (TGA), scanning electron microscopy with energy-dispersive X-ray spectroscopy (SEM-EDX), and gas adsorption porosimetry. This study uniquely evaluates their synergistic effects on hydration kinetics, compressive strength development (achieving substantial strength increases compared to ordinary Portland cement at standard testing ages), porosity refinement, and microstructural evolution. Our findings highlight the potential of these materials in low-carbon cement applications, contributing to eco-efficient construction solutions while maintaining or enhancing performance characteristics crucial for structural applications.

## 2. Materials and Methods

The materials used in this study include PC (type I), calcined pozzolans derived from kaolinite (MK), and pyroclastic rock (CPR), both commercially sourced from Grupo Cementos Chihuahua (GCC, Chihuahua, Mexico), and distilled water. The chemical composition of precursor materials was determined using a Panalytical Epsilon 3XLE energy-dispersive X-ray fluorescence spectrometer (Malvern Panalytical, Almelo, The Netherlands) (Table 1). 

The calcined natural pozzolans meet the chemical and physical requirements delineated in the ASTM C618 standard, including pozzolanic activity indices at 7 days that exceed 100% for MK and 90% for CPR, as determined according to the ASTM C311 testing protocol.

**Figure 1:**
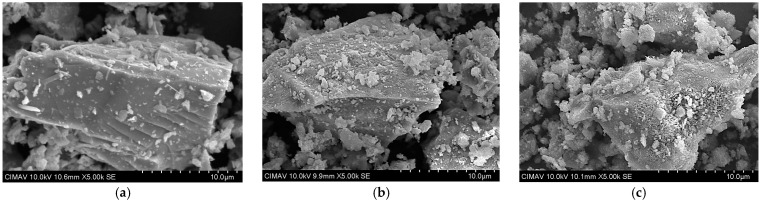
Secondary electron SEM micrographs of precursor materials: (**a**) PC, (**b**) MK, and (**c**) CPR.

Morphological characterization of precursor materials was conducted using a HITACHI SU3500 scanning electron microscope (Hitachi High-Technologies Corporation, Tokyo, Japan)) operating at 10 kV. The resulting micrographs (Figure 1) revealed angular particle morphologies characteristic of materials subjected to calcination, with particle sizes ranging from 1 to 25 μm. These angular features from pozzolans result from the mechanical grinding process, which enhances pozzolanic activity by increasing the surface area available for reaction.

**Figure 2:**
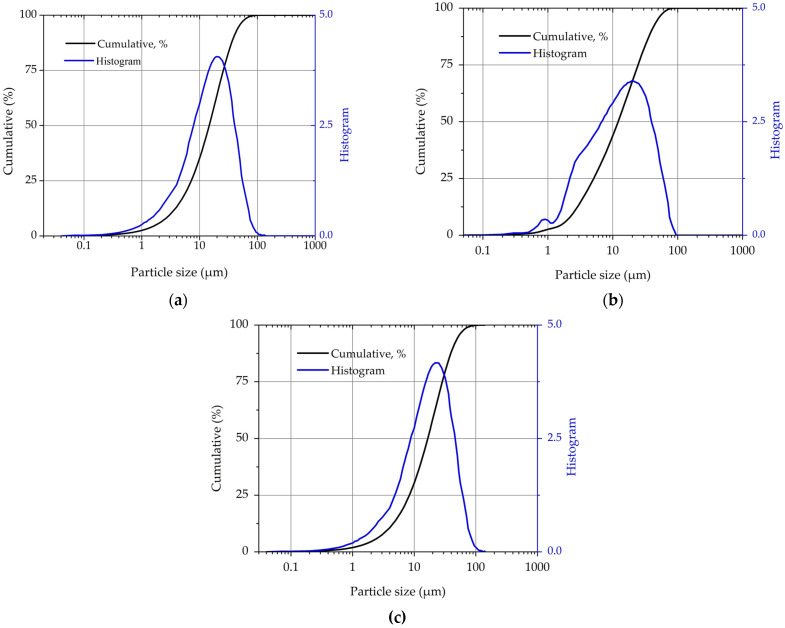
Particle size analysis of precursor materials (**a**) PC, (**b**) MK, and (**c**) CPR.

In addition, particle size analysis was performed using CILAS 1190L laser granulometry equipment (CILAS, Orléans, France) (Figure 2), which determined average particle sizes of 15 μm for PC, 12 μm for MK, and 16 μm for CPK. These morphological characteristics demonstrate the suitability of these materials for incorporation into cementitious matrices, as particle size and shape contribute to the filler effect and pozzolanic reactivity, ultimately enhancing the mechanical properties and durability of the resulting cement-based materials.

**Figure 3:**
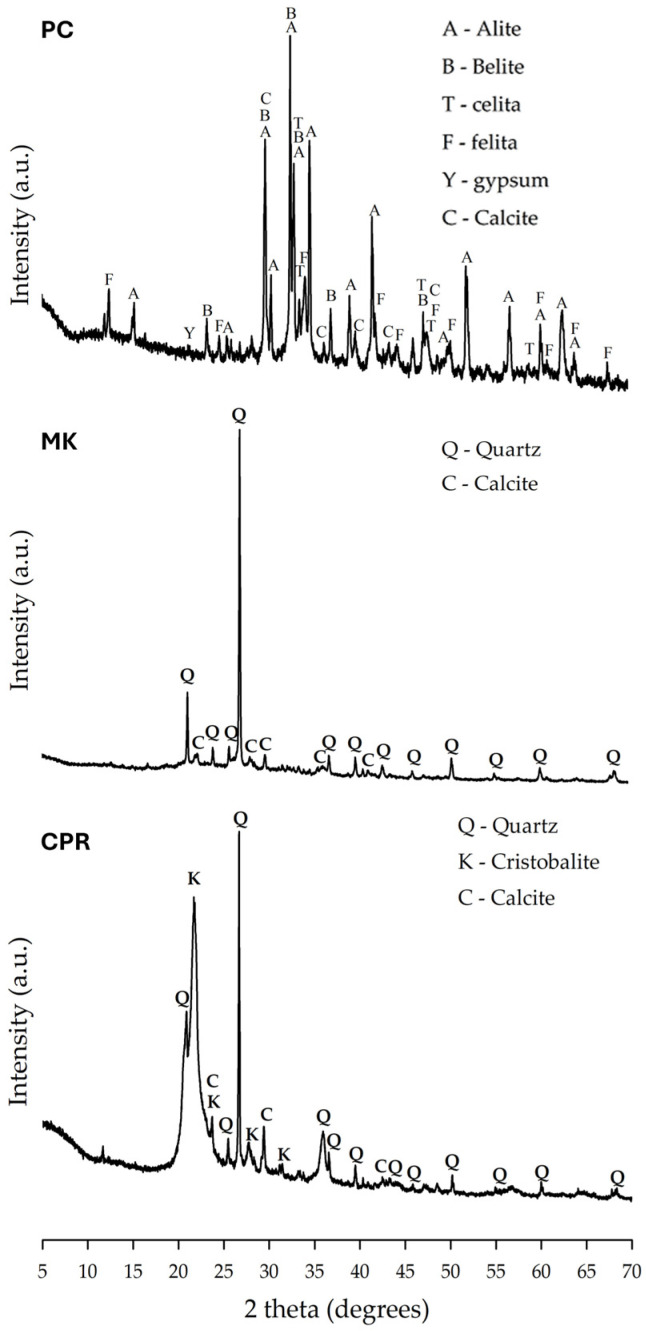
XRD patterns of precursor materials: PC, MK, and CPR.

Mineralogical composition analysis of the pozzolans was performed using a Bruker D8 Advance X-ray diffractometer (Bruker Corporation, Billerica, MA, USA) with CuKα radiation (λ = 1.5408 Å). The X-ray diffraction patterns (Figure 3) exhibit an amorphous halo between 17° and 30° 2θ, indicating the presence of an amorphous phase typical of pozzolanic materials [29]. Additionally, sharp crystalline peaks corresponding to quartz (Q) and cristobalite (K) phases, which are forms of SiO_2_, were identified. The calcite phase (C), likely a residue from the calcination process or inherent to the raw materials, was also detected. These findings confirm the dual-phase composition of the pozzolans, comprising both amorphous and crystalline components, which play a key role in their pozzolanic reactivity and performance as SCMs.

Cement paste mixtures were designed to assess the effect of calcined natural pozzolans on hydration, mechanical properties, and microstructure. The mixtures included a control paste (CP), pastes with 20% MK (K20) or CPR (P20), a 20% blended replacement (T20), and a low-carbon paste (T40) (Table 2). The T40 mixture, containing 40 wt.% pozzolan content, was included to investigate the performance capabilities of a low-carbon cementitious system incorporating calcined natural pozzolans and to determine whether higher substitution levels could maintain adequate hydration mechanisms and mechanical behavior, directly addressing contemporary sustainability imperatives. All formulations were prepared with a constant water-to-binder ratio of 0.32 and were mixed according to the protocols outlined in the ASTM C305 standard [30]. The resultant pastes were cast into 50 mm molds (three cubes per test), covered to prevent moisture loss, and subjected to controlled curing in a saturated CH solution until testing.

Setting times of the cement pastes were evaluated following the precise methodology outlined in the ASTM C191 standard [31]. Sample preparation followed the protocols specified in ASTM C305 and ASTM C187 standards [32]. In this procedure, cement pastes of normal consistency were prepared and molded into a standardized conical ring positioned on a non-absorptive plate. Periodic penetration tests were conducted using a calibrated 1 mm diameter Vicat needle. The initial setting time was recorded when the needle penetrated 25 mm into the paste matrix, while the final setting time was documented when the needle no longer produced a complete circular impression on the specimen surface.

Compressive strength evaluations were conducted in accordance with the ASTM C109 standard protocol [33], utilizing triplicate specimens cast in 50 mm cube molds. The mixing sequence and sample preparation methodologies rigorously adhered to the ASTM C305 standard to ensure specimen uniformity and data reliability. Each cubic mold was systematically filled in two distinct layers, with each layer methodically compacted using 32 uniformly distributed strokes of a standardized tamper, precisely as mandated by the standard. Following the molding process, all specimens underwent initial curing within their molds for a 24 h period, after which they were carefully demolded and immediately transferred to saturated limewater immersion environments until reaching the designated testing ages. Compression testing was performed at both 3 and 28 days of curing using a calibrated ELE International hydraulic press.

Selected fragments from the 28-day compressive strength test specimens were carefully preserved for comprehensive hydration, porosity, and microstructural analyses. To arrest ongoing hydration reactions, these representative fragments were immersed in isopropyl alcohol for 24 h and subsequently subjected to controlled drying at 60 °C for an additional 24 h. For characterization via XRD and TGA, samples were reduced to fine powder using an agate mortar and pestle and then sieved through a 75 µm mesh to ensure optimal particle fineness and sample homogeneity for instrumental analysis.

XRD was used to identify anhydrous and hydrated phases, assessing the impact of calcined natural pozzolans on hydration and microstructure. Phase indexing was conducted using X’Pert HighScore Plus software.

TGA was performed using an SDT Q600 instrument (TA Instruments, New Castle, DE, USA) under an argon atmosphere, with a heating rate of 10 °C/min up to 900 °C. Mass losses from hydrated product decomposition provided insights into the influence of calcined natural pozzolans on hydration.

Physical gas adsorption was utilized for porosity analysis, including specific surface area, pore volume, and average pore size, using a Quantachrome NovaWin system. (manufacture, city, abbreviated state (Quantachrome Instruments, Boynton Beach, FL, USA). Samples were degassed at 250 °C for 12 h. The Brunauer–Emmett–Teller (BET) model determined the specific surface area, while Barrett–Joyner–Halenda (BJH) analysis was used for pore size and volume determination.

SEM analysis was conducted using a HITACHI SU3500 microscope. Samples were gold-coated for conductivity and analyzed at 5.0 kV. Secondary electron (SE) imaging captured microstructural images at up to 10,000× magnification, enabling detailed observation of particle morphology and the cementitious matrix. EDX (Oxford Instruments, Abingdon, Oxfordshire, UK) was performed to obtain elemental composition, facilitating the identification of key hydration phases and pozzolanic reaction products within the microstructure.

The integrated application of these techniques provided a comprehensive understanding of hydration, mechanical properties, porosity, and microstructure, highlighting the impact of MK and CPR, both individually and in combination, as pozzolanic materials for low-carbon cement development.

## 3. Results and Discussion

### 3.1. Setting Time

The setting time results highlight the influence of calcined natural pozzolans on hydration kinetics in cementitious systems (Table 3). The initial setting time marks the point at which cement paste loses plasticity, while the final setting time denotes the start of hardening and initial strength development [34]. The reduction in initial setting time observed in all pozzolan-containing pastes (3–16 min) suggests an acceleration in early hydration reactions, primarily attributed to the nucleation sites provided by finely divided pozzolanic particles [35,36]. This is supported by the particle size analysis shown in Figure 2, where MK exhibits a smaller average particle size compared to PC, while CPR exhibits a comparable distribution profile, both materials possessing a significant fine fraction capable of effectively promoting nucleation processes. In contrast, the variations in final setting times are predominantly influenced by the specific replacement level. Higher replacements, such as the T40 mixture, increase clinker dilution and water demand (w/b = 0.32), which consequently decelerates later-stage hydration mechanisms and extends the setting time duration [37].

These results confirm that CPR, either alone or combined with MK, meets practical setting time requirements and can effectively replace 20–40 wt.% of PC in cementitious systems. The T20 paste, with a final setting time of 219 min (only 11 min shorter than CP at 230 min), exhibited behavior very similar to the control, indicating that moderate pozzolan incorporation accelerates early hydration without significantly altering the hardening process. Similarly, the T40 paste (low-carbon cement) had a final setting time of 255 min (+25 min compared to CP), primarily due to its higher water demand (w/b = 0.32) and reduced availability of clinker hydration products. Despite these variations, the setting time differences between pozzolan-containing pastes and the control remained relatively small, ensuring that clinker replacement does not compromise construction efficiency.

As a critical parameter in concrete construction, setting time directly affects material performance, formwork removal, and washout prevention. These findings demonstrate that optimizing pozzolan content provides control over setting characteristics, balancing hydration kinetics and early strength development. This reinforces the feasibility of using calcined natural pozzolans in low-carbon cement, reducing clinker content while maintaining performance comparable to conventional cement in construction applications.

### 3.2. Compressive Strength

**Figure 4:**
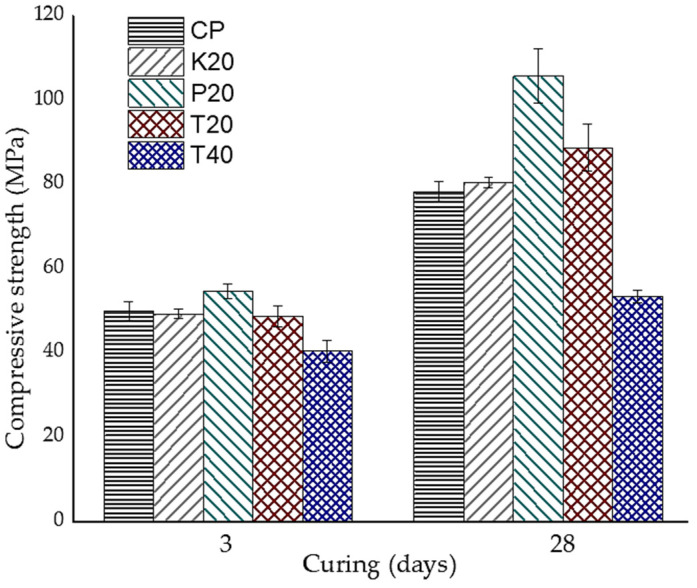
Compressive strength development of samples at 3 and 28 days of curing.

Figure 4 presents the compressive strength results of the studied pastes at 3 and 28 days of curing, with error bars indicating standard deviation values from triplicate specimen testing. At 3 days of curing, the paste containing 20 wt.% CPR (P20) exhibited the highest compressive strength, reaching 54.7 MPa. This superior early-age strength enhancement can be attributed to the high SiO_2_ content of CPR (Table 1), which actively promotes accelerated pozzolanic reactions with CH, coupled with the predominantly amorphous nature of the material, evidenced by the distinctive broad halo observed in its XRD pattern (Figure 3). These properties facilitate the formation of secondary hydration products such as C-S-H, significantly contributing to early-age strength development [38,39]. These findings highlight the potential of CPR as a promising SCM for enhancing hydration kinetics and mechanical performance in blended cement systems.

The K20 and T20 pastes achieved comparable compressive strength values, with only 1.2% and 2.4% lower strength than the control paste, respectively. The T40 paste exhibited a 19% reduction in compressive strength compared to the control. However, considering that this mixture exceeds the typical recommended replacement level for calcined clays (approximately 20 wt.%) [40], the observed strength values still support its feasibility for low-carbon cement applications.

At 28 days of curing, the P20 paste demonstrated superior performance, reaching 105.7 MPa—the highest value among all mixtures—and exceeding the control paste (CP) by 35.2%. This result can be attributed to the pozzolanic reactivity of CPR with CH, which refined the pore structure [41]. Although the T20 mixture showed lower strength compared to P20, it achieved a compressive strength of 88.7 MPa, representing a 13.5% improvement over the control paste. These results highlight the potential of combining calcined natural pozzolans to optimize the mechanical properties of cement systems.

The T40 paste, with a 40 wt.% replacement level, achieved a compressive strength of 53.5 MPa, which, although lower than the control paste (78.2 MPa), aligns with expected performance for cementitious systems incorporating high pozzolan replacement levels. The strength reduction primarily results from clinker dilution; however, continued pozzolanic reactions are expected to enhance long-term performance by promoting secondary hydration products, leading to pore structure refinement, reduced permeability, and improved durability. These characteristics position T40 as a viable alternative for low-carbon cement applications, particularly in scenarios prioritizing sustainability and long-term material performance.

The results prominently demonstrate CPR’s potential as an SCM for low-carbon cement. Its higher pozzolanic activity enables greater clinker replacement levels compared to MK, while simultaneously achieving the highest compressive strengths among the studied pozzolanic systems. The incorporation of CPR into cementitious systems not only enhances mechanical performance but also significantly reduces the carbon footprint, aligning with global sustainability objectives in the construction industry. These findings reinforce CPR’s role in advancing low-carbon cement formulations, offering an optimal balance between strength development, durability, and environmental impact reduction.

### 3.3. X-Ray Diffraction

**Figure 5:**
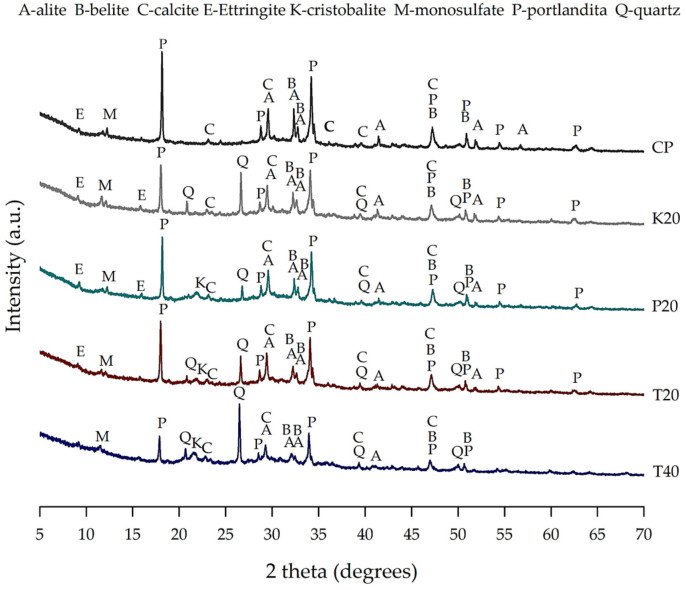
XRD patterns of samples at 28 days of curing.

Figure 5 presents the XRD patterns of the control paste and pastes containing MK, CPR, and their combinations (T20 and T40) after 28 days of curing. The dominant crystalline phase in all pastes is portlandite, a primary hydration product typically accounting for 20–25% of the solid volume in systems without SCMs. In pozzolan-containing pastes, the reduced CH peak intensity indicates its consumption through pozzolanic activity, resulting in the formation of poorly crystalline phases, such as C-S-H and C-A-S-H, as well as crystalline calcium aluminate hydrates (e.g., C_4_AH_13_ and C_3_AH_6_) in MK-containing pastes. The amorphous hump in the 2θ range of 25°–40° corresponds to these poorly crystalline phases, highlighting the microstructural refinement and enhanced durability achieved by incorporating calcined natural pozzolans [42].

The XRD patterns confirm the presence of ettringite (E) and monosulfate (M) in all mixtures, with characteristic diffraction peaks at 2θ ≈ 9.1° and 2θ ≈ 11.6°, respectively. These phases, formed through the reaction between aluminates and gypsum, play a crucial role in early microstructural stabilization and hydration kinetics. Additionally, residual anhydrous phases, including quartz (Q), cristobalite (K), calcite (C), alite (A), and belite (B), were identified, with primary diffraction peaks at 2θ ≈ 26.6° (Q), 21.9° (K), 29.4° (C), 32.2° (A), and 29.5° (B). The reduced peak intensities of these phases, compared to their raw material counterparts, indicate progressive hydration and continued phase transformations. In the T40 mixture, the higher intensity of quartz peaks reflects the greater pozzolan content, as quartz is the dominant crystalline phase in these materials, as previously observed in the XRD patterns of the precursor materials (Figure 3). Furthermore, the ongoing hydration process at 28 days suggests that these peaks may diminish at later ages, as unreacted cementitious and pozzolanic particles continue to participate in hydration reactions, contributing to further microstructural development.

The presence of residual anhydrous phases can be attributed to the particle size distribution of Portland cement and pozzolans. Smaller particles hydrate rapidly, while larger particles hydrate more slowly and can continue reacting over extended periods, even beyond 28 days, provided favorable moisture conditions are maintained [43]. This behavior is characteristic of cementitious systems and highlights the ongoing reactivity potential of anhydrate phases under prolonged curing.

In summary, the XRD analysis at 28 days of curing confirms the potential of calcined natural pozzolans derived from pyroclastic rock and thermal activation as eco-efficient SCMs for low-carbon cement production. Their ability to reduce CH content while promoting the formation of secondary hydration products underscores their viability as a sustainable alternative to traditional SCMs, particularly in volcanic regions. The presence of residual anhydrous phases and unreacted CH suggests that hydration will continue over time, provided that favorable moisture conditions are maintained, further enhancing the long-term performance of the system.

### 3.4. Thermogravimetric Analysis

**Figure 6:**
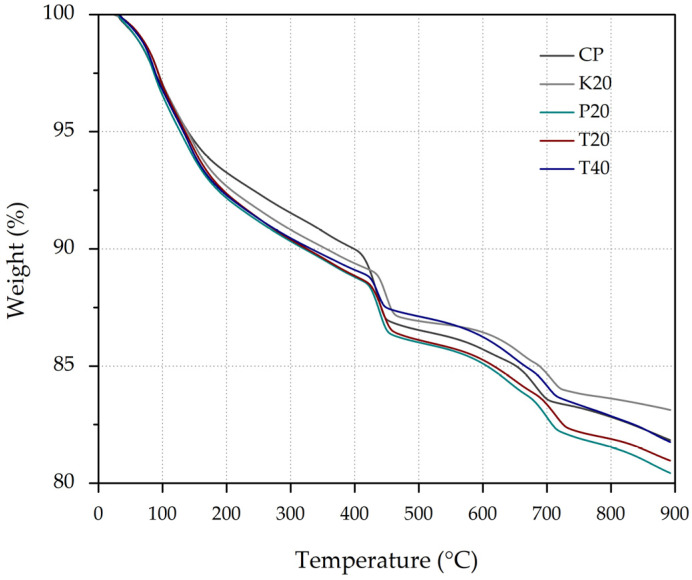
Thermogravimetric analysis of the samples at 28 days of curing.

The thermogravimetric analysis results for the studied cement pastes at 28 days of curing are presented in Figure 6. The thermal decomposition profiles exhibit well-defined mass loss regions, each associated with specific dehydration and decomposition processes. These patterns offer valuable insights into phase composition and hydration product development. Table 4 provides a detailed summary of the mass loss percentages observed across each temperature region for the different samples, facilitating a comparative assessment of their thermal behavior.

The first mass loss, occurring in the range of ambient temperature to 110 °C, is associated with the evaporation of free and loosely bound water within the pastes’ pore structure. This initial dehydration stage reflects both the physically adsorbed water and the water contained within gel pores, serving as an indicator of the pastes’ moisture retention capacity. Notably, the T20 and T40 pastes exhibit slightly higher mass losses in this range, suggesting enhanced water retention within their refined pore networks.

The second mass loss, within the 110–400 °C range, corresponds to the dehydration of chemically bound water from primary hydration products. This region predominantly reflects the decomposition of C-S-H, C-A-S-H, and C-A-H phases [44]. The pastes containing pyroclastic rock (P20 and T20) demonstrate significantly higher mass losses in this temperature range compared to both the control paste and K20, indicating a greater quantity of hydrated products. This observation aligns with the compressive strength results presented in Section 3.2, where these mixtures displayed superior mechanical performance. The enhanced mass loss can be attributed to the highly reactive amorphous silica content in CPR, which accelerates and amplifies pozzolanic reactions, resulting in the formation of additional secondary hydration phases with stronger binding properties [45]. Quantitatively, the P20 paste shows approximately 13% higher mass loss in this region compared to the control, correlating well with its 35.2% higher compressive strength at 28 days of curing.

The third mass loss region, observed between 400 °C and 500 °C, is attributed to the dehydroxylation of portlandite (CH). This temperature range serves as a critical indicator of pozzolanic reactivity, as it quantifies the residual CH content after potential consumption by SCMs. The control paste exhibits the highest mass loss in this range (approximately 3.7%), consistent with its higher CH content observed in the XRD analysis. In contrast, pozzolan-containing pastes show progressively reduced mass losses in this region, with the T40 paste demonstrating the lowest value (approximately 2%), representing a 45% reduction compared to the control. This substantial decrease confirms the enhanced pozzolanic activity of natural calcined pozzolans, which effectively consumes CH to form additional C-S-H and other binding phases [46].

The final significant mass loss, occurring between 600 °C and 700 °C, is associated with the decarbonation of calcium carbonate (CaCO_3_), predominantly in the form of calcite [47]. The presence of calcite in these systems can arise from various sources, including the carbonation of CH during sample preparation, residual carbonate from the precursor materials, or environmental exposure. This interpretation is further supported by the XRD patterns of all precursor materials (Figure 3), including PC, which distinctly exhibit calcite peaks.

When analyzing the overall thermal behavior, the pozzolan-containing pastes demonstrate a marked shift in decomposition patterns, characterized by reduced CH content and increased hydration product formation. The integration of the TG curves reveals that the P20 paste exhibits the highest total mass loss associated with hydration products (approximately 12%). This finding conclusively demonstrates the superior hydration efficiency achieved through pyroclastic rock incorporation, either individually or in combination with calcined kaolin.

In summary, the TGA results provide quantitative evidence of enhanced hydration kinetics and phase transformation processes in pastes containing calcined pyroclastic rock. These findings align with the compressive strength and XRD results, confirming that pastes containing CPR and ternary blends exhibit accelerated hydration, reduced CH content, and increased formation of binding phases due to enhanced pozzolanic reactions. The molecular-level transformations observed through thermal analysis help explain the macroscopic performance improvements, highlighting the potential of calcined pyroclastic rock as a high-reactivity SCM for sustainable construction materials and its pivotal role in advancing low-carbon cement systems.

### 3.5. Physical Adsorption of Gases 

The gas adsorption results provide valuable insight into the pore structure characteristics of the cement pastes, revealing important correlations between microstructural refinement and both durability and mechanical performance. Table 5 presents a comprehensive comparison of average pore size and pore volume measurements across all mixture formulations at 28 days of curing.

The results demonstrate that pastes incorporating calcined pyroclastic rock (P20) and its combination with calcined kaolin (T20) exhibit significantly more refined pore structures compared to the control paste (CP). The T20 formulation achieves the smallest average pore size (7.534 nm), representing a 7.5% reduction relative to the control paste (8.143 nm), while P20 follows closely with an average pore size of 7.965 nm. This pore size refinement directly contributes to the enhanced mechanical properties observed in these mixtures, as smaller pore sizes generally correspond to improved strength and durability characteristics.

The pore volume measurements reveal complementary information about microstructural development. While P20 shows a slightly higher pore volume (0.044 cm^3^/g) compared to CP (0.040 cm^3^/g), this increase is offset by the significant reduction in average pore size, resulting in a more refined pore network that effectively distributes porosity throughout the matrix. This optimization of pore structure contributes to the superior compressive strength observed in P20 samples at 28 days of curing, as demonstrated in Section 3.2.

In contrast, the K20 formulation exhibits the largest average pore size (8.365 nm) among all mixtures but the lowest pore volume (0.037 cm^3^/g). This unique combination suggests a more compact but less refined pore structure, where fewer but larger pores dominate the microstructure. The reduced pore volume indicates a densification effect, but the presence of larger pores may limit the efficiency of hydration product formation and distribution compared to pyroclastic rock-containing formulations.

The T40 mixture, with its higher pozzolan replacement level (40 wt.%), presents an intermediate average pore size (8.013 nm) that is still smaller than the control paste. However, it exhibits the highest pore volume (0.064 cm^3^/g) among all formulations, reflecting its higher clinker dilution and increased water demand. This characteristic suggests ongoing hydration processes within the matrix, with continued pore refinement expected over extended curing periods as secondary hydration products continue to develop and fill the available pore space.

The porosity results align closely with the hydration kinetics observed in thermogravimetric analysis (Section 3.4), where pastes containing pyroclastic rock demonstrated enhanced formation of secondary hydration products. These products, primarily C-S-H and C-A-S-H gels, contribute to pore refinement by filling capillary pores and creating a more homogeneous microstructure. The superior pore structure refinement of T20 and P20 formulations directly correlates with their enhanced mechanical performance, confirming the effectiveness of these calcined natural pozzolans in optimizing the microstructural characteristics of low-carbon cement systems.

These findings highlight the potential of calcined natural pozzolans in developing sustainable cementitious materials with optimized pore structures while significantly reducing clinker content. The ability to achieve comparable or superior microstructural refinement with reduced clinker content reinforces the viability of these materials for low-carbon cement applications, particularly in contexts where environmental impact reduction is prioritized alongside mechanical performance.

### 3.6. Scanning Electron Microscopy

**Figure 7:**
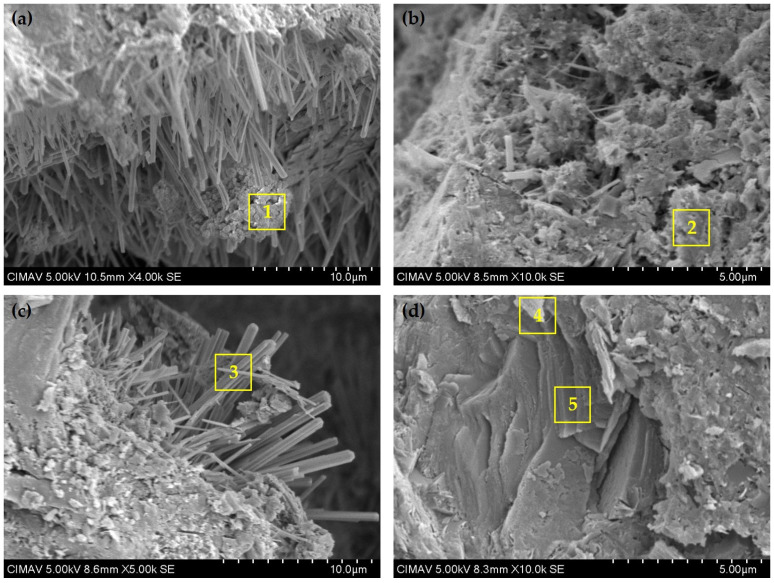
SEM micrographs of samples at 28 days of curing: (**a**) CP, (**b**) K20, (**c**) T20, and (**d**) T40 formulations.

Figure 7 presents SEM micrographs of CP, K20, T20, and T40 pastes after 28 days of curing, revealing distinct microstructural features across formulations. These images capture a heterogeneous cementitious matrix characterized by diverse hydration products including C-S-H, ettringite, C-A-S-H, and C-A-H, as well as unreacted particles of pozzolans and cement clinker. The morphological characteristics vary considerably depending on paste composition, with higher pozzolan replacement levels exhibiting more pronounced development of laminar and acicular phases. Table 6 provides complementary EDX elemental analysis of specific microstructural zones, establishing quantitative correlations between observed morphologies and chemical compositions of the identified phases in each formulation.

In Figure 7a, corresponding to the control paste (CP), a relatively dense but inhomogeneous microstructure is evident, dominated by characteristic needle-like ettringite crystals (3CaO·Al_2_O_3_·3CaSO_4_·32H_2_O) arranged in radial clusters throughout the hydrated matrix. These distinctive formations, typically 2–10 µm in length, indicate advanced hydration processes and are embedded within a surrounding matrix of C-S-H gel [48]. The high calcium-to-silicon ratio observed in Zone 1 (Ca: 23.9%, Si: 7.6%, Table 6) confirms the predominance of PC hydration products with limited pozzolanic modification. The presence of sulfur (1.4%) further supports the identification of ettringite formations within this zone.

The K20 formulation (Figure 7b) exhibits a notably different microstructural arrangement, characterized by a significant reduction in visible ettringite crystals and the development of a more homogeneous, densified matrix. This transformation suggests active pozzolanic reactions between MK and PC, leading to the formation of C-A-S-H gel and additional secondary C-S-H phases. The increased silicon and aluminum content detected in Zone 2 (Si: 8.7%, Al: 3.2%, Table 6) compared to Zone 1 provides quantitative evidence of these pozzolanic reaction products. This refined microstructure contributes to enhanced material density and reduced permeability, which correlates with the improved durability characteristics commonly observed in kaolin-modified cementitious systems.

The T20 formulation (Figure 7c) presents a microstructure that reflects the synergistic effects of both pozzolanic materials, combining features observed in both CP and K20 samples. The presence of well-defined hydrated products, including distinctive ettringite formations alongside a more uniform distribution of C-S-H and C-A-S-H gels, indicates complementary contributions from both supplementary cementitious materials. Zone 3, featuring prominently visible acicular structures, shows a chemical composition highly characteristic of ettringite (Ca: 34.7%, S: 6.1%, Al: 3.5%, Si: 1.6%), with the higher sulfur content definitively distinguishing these formations from other hydration products. The elemental profile and distinctive needle-like morphology provide conclusive identification of these ettringite crystals, which play an important role in early strength development and microstructural stabilization.

The T40 formulation (Figure 7d) likely exhibits the most pronounced microstructural modifications due to its higher pozzolan replacement level (40 wt.%). The visual examination suggests a more heterogeneous matrix with a greater prevalence of unreacted pozzolanic particles embedded within developing hydration products. Zone 4 corresponds to a dense, angular particle with a distinctive compositional profile (Ca: 26.7%, Si: 7.0%, Al: 5.0%, Fe: 5.4%), suggesting a combination of partially hydrated clinker phases alongside developing C-A-S-H phases. The elevated iron content particularly distinguishes this zone, indicating potential ferrite phase contributions to the developing microstructure. Meanwhile, Zone 5 exhibits a characteristic laminar morphology with significantly higher aluminum content (Al: 9.1%) coupled with relatively low silicon content (Si: 0.7%), strongly indicative of C-A-H phases. This compositional profile, combined with the distinctive plate-like structure, confirms the substantial influence of calcined kaolin on the formation of alumina-rich hydration products, which modify both the microstructural arrangement and mechanical properties of the cementitious matrix.

These microstructural findings conclusively demonstrate that incorporating calcined natural pozzolans significantly modifies the hydration pathway in cementitious systems, shifting from ordinary Portland cement hydration products toward more diverse phase assemblages including additional C-S-H, C-A-S-H, and C-A-H formations. At higher pozzolan replacement levels, the microstructure progressively transitions toward alumina-rich phases, directly influencing porosity characteristics, mechanical properties, and long-term durability performance in these low-carbon cement systems. The microscopic evidence presented here provides a direct visualization of the mechanisms underlying the enhanced performance observed in macroscopic testing, reinforcing the viability of these sustainable material formulations for advanced construction applications.

## 4. Conclusions

Incorporating calcined natural pozzolans, such as CPR and MK, into cementitious systems enhances hydration kinetics, mechanical performance, and microstructural refinement, offering eco-efficient alternatives to ordinary Portland cement and reducing clinker demand and CO_2_ emissions.

Moderate replacement levels (20 wt.%) of these pozzolans accelerate the hydration process without adversely affecting setting times, making them suitable for practical concrete applications.

The paste containing 20 wt.% CPR (P20) exhibited superior compressive strength at both 3 and 28 days of curing, outperforming the control sample. This enhancement is attributed to CPR’s high pozzolanic reactivity, which facilitates the formation of additional C-S-H phases that strengthen the cementitious matrix.

Similarly, the ternary blend (T20) surpassed the control sample in 28-day compressive strength, benefiting from the synergistic effects of combining MK and CPR, which optimizes hydration reactions and microstructural development more effectively than either pozzolan alone.

While higher replacement levels (40 wt.%) achieved somewhat lower strengths than the control paste, the values remained well within acceptable parameters for structural applications, with ongoing hydration expected to enhance long-term performance through progressive refinement of the pore structure.

XRD and TGA analyses confirmed that incorporating MK and CPR leads to reduced CH content and subsequent formation of beneficial secondary hydration products, including C-S-H, C-A-S-H, and C-A-H. These products, directly observed through SEM-EDX analysis, contribute significantly to microstructural refinement and improved mechanical properties.

Detailed porosity assessments revealed that the K20 mixture exhibits the lowest overall pore volume, while CPR-containing formulations (P20, T20, and T40) demonstrated reduced average pore sizes compared to the control mixture, indicating a more refined microstructure with enhanced durability potential.

The strategic combination of CPR with MK enhances hydration kinetics, mechanical strength, and microstructural refinement more effectively than blends with MK alone or the control paste, ensuring adequate performance for sustainable construction applications.

CPR emerges as a promising material for developing low-carbon cement and high-performance cement-based composites due to its notable pozzolanic reactivity, widespread availability in volcanic regions, and significantly lower processing energy requirements compared to conventional clinker production.

While these findings demonstrate significant improvements with CPR incorporation, further comprehensive durability assessments under diverse environmental exposure conditions remain essential to fully validate the long-term performance of these materials in low-carbon cement formulations for sustainable construction.

## Figures and Tables

**Table 1:** Chemical composition of precursor materials.

Material	SiO_2_	Al_2_O_3_	Fe_2_O_3_	CaO	MgO	Na_2_O	K_2_O	SO_3_
PC	19.28	4.93	3.52	63.76	1.80	0.10	0.53	2.36
MK	54.77	26.15	1.33	8.01	0.54	0.00	0.19	1.57
CPR	79.26	6.50	1.64	5.65	0.15	0.00	0.11	1.28

**Table 2:** Mixture proportions of cement pastes (%).

Mix	PC	MK	CPR
CP	100	0	0
K20	80	20	0
P20	80	0	20
T20	80	10	10
T40	60	25	15

**Table 3:** Setting time (min) of samples.

Mix	Initial	Final	w/b
CP	127	230	0.26
K20	124	167	0.28
P20	115	173	0.30
T20	113	219	0.29
T40	111	255	0.32

**Table 4:** Mass loss percentages in different temperature ranges for the analyzed pastes.

Paste	25–110 °C (%)	110–400 °C (%)	400–500 °C (%)	600–700 °C (%)
CP	3.72	7.75	3.66	2.73
K20	3.12	7.84	2.36	2.82
P20	3.80	8.74	2.87	1.81
T20	4.49	8.33	2.63	2.27
T40	4.72	8.11	2.02	1.46

**Table 5:** Average size and pore volume of the pastes at 28 days of curing.

Paste	Average Pore Size (nm)	Pore Volume (cm^3^/g)
CP	8.143	0.040
K20	8.365	0.037
P20	7.965	0.044
T20	7.534	0.042
T40	8.013	0.064

**Table 6:** EDX analysis of present elements in specific zones of cement paste with pozzolans.

Zone	O	Ca	Si	Al	S	Fe
1	61.5	23.9	7.6	2.0	1.4	0.9
2	57.7	26.1	8.7	3.2	0.9	0.8
3	53.7	34.7	1.6	3.5	6.1	0.0
4	50.5	26.7	7.0	5.0	0.9	5.4
5	58.6	28.7	0.7	9.1	0.6	2.0

## Data Availability

The original contributions presented in this study are included in the article. Further inquiries can be directed to the corresponding authors.
